# High VCP/p97 expression associates with low PD-L1 TPS in oropharyngeal squamous cell carcinoma

**DOI:** 10.1007/s12672-025-03839-8

**Published:** 2025-10-20

**Authors:** Inga Marte Charlott Seuthe, Markus Ruwe, Franz Mitze, Steffi Silling, Eric Ehrke-Schulz, Sabine Eichhorn, Jonas Jae-Hyun Park

**Affiliations:** 1 Department of Otorhinolaryngology, Head and Neck Surgery, University of Witten/Herdecke, Catholic Hospital Hagen, Dreieckstraße 15, 58097 Germany Hagen,; 2 Institute for Pathology Hagen, Buscheystr. 15, 58089 Hagen, Germany; 3 Institute of Virology, National Reference Center for Papilloma- and Polyomaviruses, Faculty of Medicine and University Hospital of Cologne, Fürst-Pückler- Straße 56, 50935 Cologne, Germany; 4 Department of Human Medicine, Faculty of Health, Center of Biomedical Education and Research (ZBAF), Institute for Virology and Microbiology, University of Witten/Herdecke, Stockumer Straße 10, Witten, 58453 Germany

**Keywords:** PD-L1, VCP, OPSCC, HNSCC, TILS

## Abstract

**Objectives:**

VCP/p97 inhibition is known to upregulate PD-L1. This study investigated the correlation between PD-L1 and VCP/p97 expression in OPSCC, as well as the expression and prognostic significance of VCP/p97 in OPSCC.

**Methods:**

The study retrospectively included 56 patients with OPSCC. Immunohistochemical staining was performed to detect VCP/p97 and PD-L1 expression. The staining intensity of VCP/p97 and PD-L1 TPS and CPS was determined. HPV status was evaluated using immunohistochemical staining against p16 and HPV PCR. In addition, iTILs were assessed.

**Results:**

High VCP/p97 expression was observed in 67.9% of the tumors. A significant association was found between high VCP/p97 expression and low PD-L1 TPS (*p* = 0.025) and between high VCP/p97 and low iTILs (*p* = 0.015). No significant correlation was found between VCP/p97 and PD-L1 CPS. VCP/p97 showed no prognostic significance in OPSCC.

**Conclusion:**

VCP/p97 is frequently overexpressed in OPSCC. The significant association of high VCP/p97 expression with low PD-L1 TPS and reduced iTILs could indicate that VCP/p97 inhibitor therapy may enhance the efficacy of PD-1 checkpoint inhibitor therapy in OPSCC.

## Introduction

The incidence of oropharyngeal squamous cell carcinoma (OPSCC) has been increasing worldwide in the past few years [34]. Major risk factors for the development of OPSCC are tobacco and excessive alcohol consumption as well as infection with human papillomaviruses (HPV) [4, 35]. Over 200 different HPV types are currently known, a number of which -primarily HPV16, 18, 31, but also 33, 35, 39, 45, 51, 52, 56, 58, 59, 68, 73 and 82- are classified as high-risk types due to their carcinogenic potential [14].

The relative 5-year survival rate in oropharyngeal squamous cell carcinoma across all tumor stages under current treatment regimens is 52% for men and 64% for women [16]. As with all cancer therapies, treatment resistance is a major limiting factor. It is therefore of crucial importance to optimize known therapeutic approaches and to develop new therapies that extend and improve the treatment of OPSCC. An approved therapy for advanced OPSCC is treatment with immune checkpoint inhibitors as monotherapy or in combination with chemotherapy [2, 13]. However, about 60% of patients with head and neck squamous cell cancer (HNSCC) show resistance to treatment with checkpoint inhibitors like programmed cell death protein 1 (PD-1)/ programmed death-ligand 1 (PD-L1) inhibitors and only 20–30% of patients show no disease progression in the long term [11]. The failure of immunotherapy is due to ubiquitous immunosuppression caused by intrinsic or adaptive resistance mechanisms of the carcinoma. For example, it is known that the expression of Toll-like receptor 4 (TLR4) protects HNSCC tumor cells from immune attack of natural killer (NK) cells through the anti-apoptotic effects of activated nuclear factor κB (NF-κB) [22]. The activation of the PI3K- pathway in response to immunotherapy enhances the expression of the immunosuppressive cytokines chemokine ligand 2 (CCL2) and vascular endothelial growth factor (VEGF) while simultaneously impairing CD8 + T cell infiltration into the tumor [15]. The pre-existing tumor environment influences responsiveness to checkpoint inhibitor treatment. For example, tumors that naturally lack antigens or T cells usually do not respond to anti-PD1 therapy [24]. Therefore, therapies that can create an immunogenic environment in tumors have the potential to provide clinical efficacy to checkpoint blockade. Agents that can induce immunogenic cell death (ICD) play a promising role here.

The highly conserved AAA-Atpase valosin containing protein (VCP/p97) plays a significant role in various cellular functions. These include endoplasmic reticulum-associated degradation (ERAD), mitochondrial-associated degradation (MAD) and the ubiquitin-proteasome system (UPS) [26]. It is known that inhibition of VCP/p97 can induce immunogenic cell death (ICD) in tumor cells [7, 28]. In a study with colon carcinomas, Wang et al. [28] showed an upregulation of PD-L1 by VCP/p97 inhibition via the JAK1/STAT3 pathway. In addition, they were able to show significantly inhibited tumor growth in the colon cancer mouse model with immunocompetent BALB/c mice in combination treatment with PD-1 inhibitor and VCP/p97 inhibitor compared to PD-1 inhibitor therapy alone. The JAK1/STAT3 pathway also plays an important role in the development of HNSCC, as it regulates the signaling cascades involved in tumor progression [10]. Various studies have already shown an increased expression of VCP/p97 in solid tumors [23, 29–33].

Nonetheless, there is currently a lack of studies investigating PD-L1 expression and intratumoural tumour infiltrating lymphocytes (iTILs) in relation to VCP/p97 in OPSCC. To date, only one study has examined VCP/p97 expression specifically in OPSCC [12].

To gain additional insights in this regard, a retrospective study of VCP/p97 expression, PD-L1 expression and iTILs was performed in a collective of OPSCC.

## Materials and methods

### Subjects and material

Tissue samples of OPSCC from 56 patients were analyzed for PD-L1 and VCP/p97 expression and iTILs. The samples were obtained either during the initial diagnostic panendoscopy or during tumor resection. No patient had undergone radiation, chemotherapy, or immunotherapy at the time of sample collection. Clinical patient data are summarized in Table 1. The tumor stage was determined according to the 8th edition of the American Joint Committee on Cancer Staging (AJCC) [1]. In most cases, patients were treated with multimodal therapy including surgical resection and adjuvant radio(chemo/immune) therapy. Follow-up was defined as the interval between initial diagnosis and either the last follow-up visit or the date of death. The median follow-up time was 57.4 months, ranging from 0.7 to 122.3 months. Tissue was fixed in 4% buffered formalin and embedded in paraffin according to routine procedures. All sections were initially reviewed by the pathologist (M.R.) after hematoxylin-eosin staining to ensure that representative tumor areas were selected for further analysis. The study was approved by the Ethics Committee of Witten-Herdecke University (ethics application number: S-242/2024) and conducted in accordance with the latest version of the Declaration of Helsinki. Informed consent was not obtained from patients due to the retrospective nature of the study. Obtaining consent would have involved disproportionate effort, particularly as many participants had already died. The requirement for written informed consent was therefore waived by the Ethics Committee of Witten-Herdecke University.

**Table 1:** Clinicopathological characteristics of the OPSCC patients

Characteristic	No. of patients	%	*N* (Total)
Sex			56
Male	42	75.0	
Female	14	25.0	
Age			
Median	61.60		
Range			
<=Median	29		
>Median	27		
T-stage			56
1	13	23.2	
2	18	32.1	
3	13	23.2	
4	12	21.4	
N-stage			56
0	21	37.5	
1	11	19.6	
2	24	42.9	
3	0	0.0	
M-stage			56
0	55	98.2	
1	1	1.8	
Grading			56
1	3	5.4	
2	24	42.9	
3	29	51.8	
4	0	0.0	
AJCC			56
I	18	32.1	
II	9	16.1	
III	8	14.3	
IV	21	37.5	
Treatment			56
Surgery + RT/RCT/RIT	25	44.6	
RT/RIT/RCT alone	14	25.0	
Surgery alone	16	28.6	
Best supportive care	1	1.8	
Regular smoking			56
+	44	78.6	
-	12	21.4	
Regular drinking			56
+	21	37.5	
-	35	62.5	
HPV status (p16 and PCR)			54
+	26	48.1	
-	28	51.9	

## Immunohistochemistry for PD-L1, VCP/p97 and p16

Tissue sections were stained using the VENTANA BenchMark ULTRA immunostainer (Roche). The immunostaining utilized the biotin-free complex method. The immunostaining for PD-L1 was performed using the Ventana PD-L1 (SP263) Assay (Roche, Cat. No. 07419821001) (ready to use) and the immunostaining for p16 was performed using CINtec p16 histology monoclonal mouse anti-human p16INK4a antibody (Roche, Cat. No. 805–4713) (ready to use) following the manufacturers’ instructions. For VCP/p97, staining was conducted using a monoclonal mouse anti-human antibody (Santa Cruz Biotechnology, Cat. No. sc-57492) at a dilution of 1:100. Detection was performed with the ultraView Universal DAB Detection Kit (Roche). Known positive tissue for each respective antibody was co-stained as a positive control. Additionally, in each staining run, the antibody dilution solution (Antibody Diluent, Roche) was applied instead of the primary antibody on one slide to serve as a negative control. The slides were classified in a blinded fashion without knowledge of the clinical-pathological data by two authors (I.S., M.R.).

**Fig. 1:**
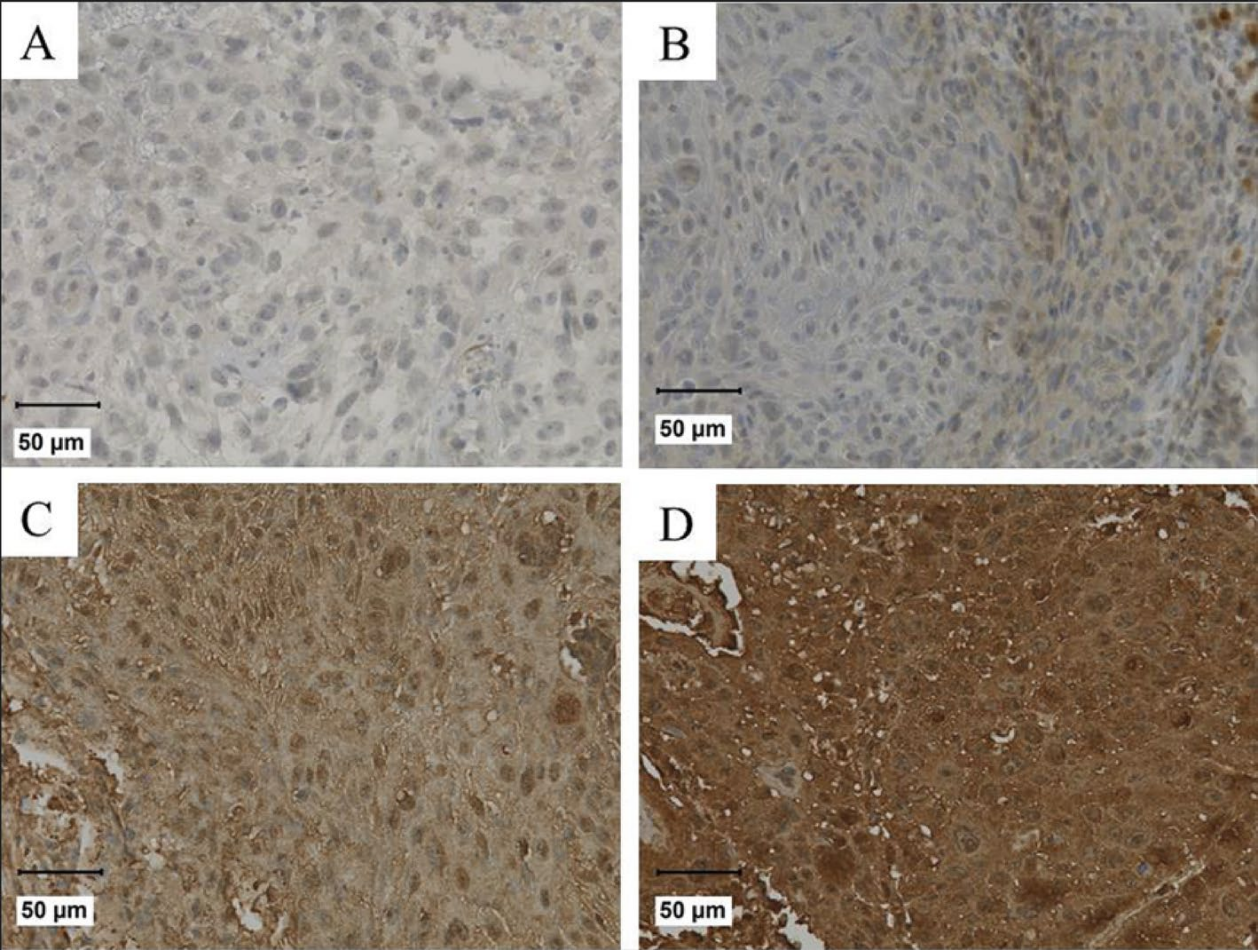
VCP-staining intensity in OPSCC patients

For VCP/p97 expression, the absolute staining intensity was evaluated and classified as follows: level 0 (without counterstaining), level 1 (low grade), level 2 (moderate grade) and level 3 (high grade) [12] (Fig. 1). In statistical analysis levels 0 and 1 were grouped as low expression, and levels 2 and 3 as high expression.

**Fig. 2:**
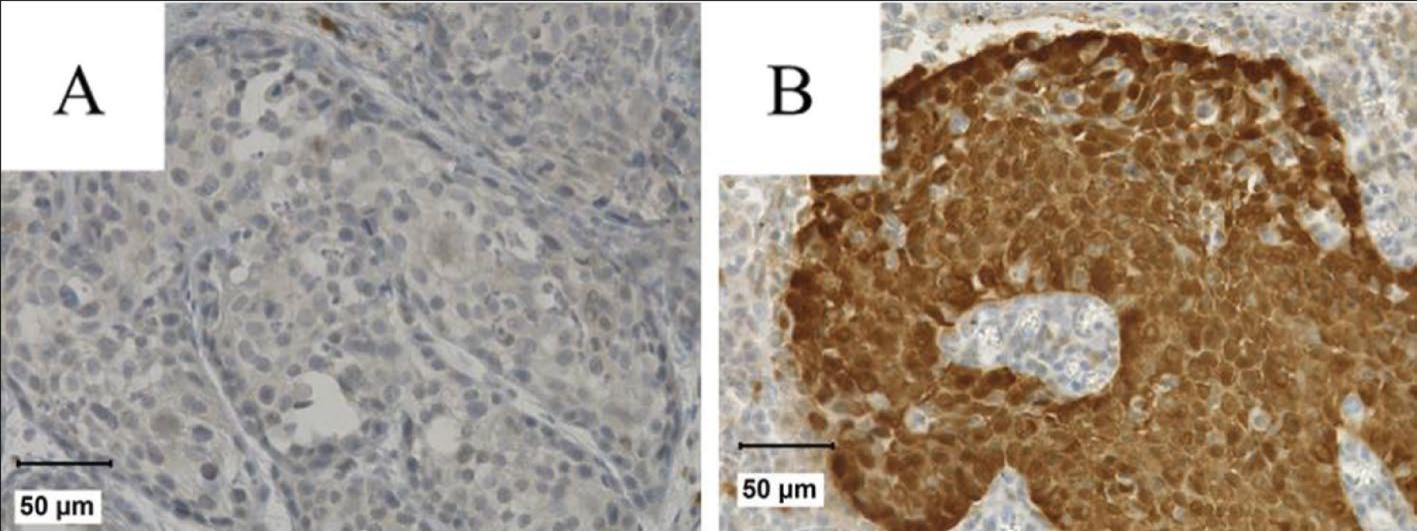
Representative p16-staining in OPSCC patients

Tumors were considered p16 positive if at least 60% of tumor cells exhibited a strong p16 staining (Fig. 2).

**Fig. 3:**
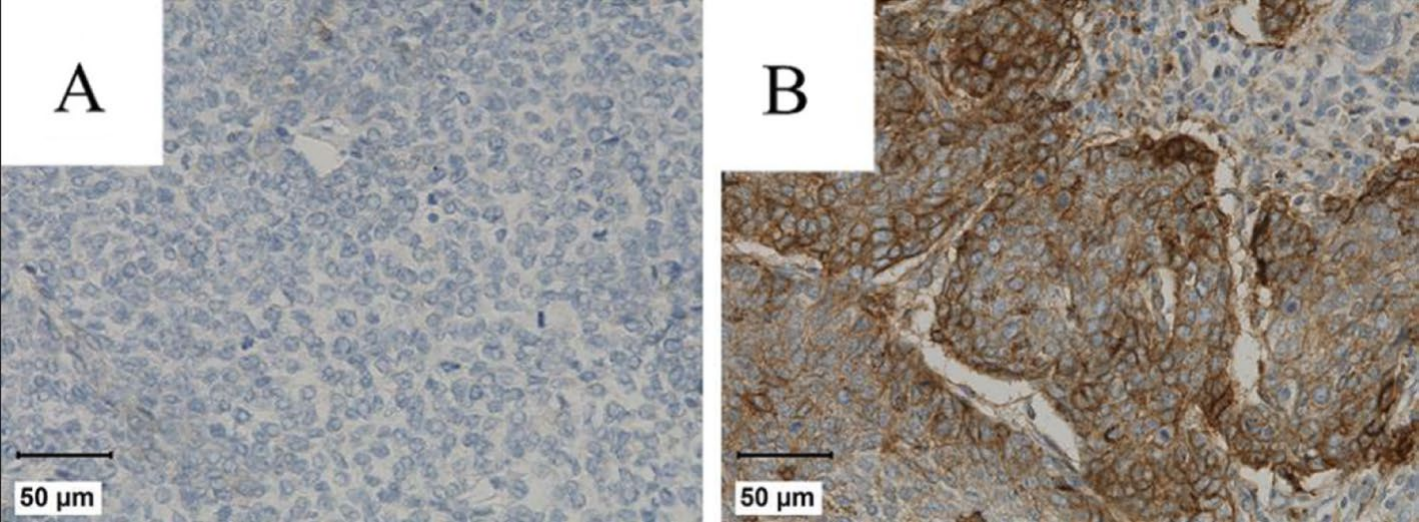
Representative PD-L1 staining in OPSCC patients

With regard to PD-L1 expression, the tumor proportion score (TPS) and the combined positivity score (CPS) were analyzed [17]. In statistical analyses, a TPS/CPS score of 0 was considered negative, scores of 1–19 were classified as low, and scores ≥ 20 were categorized as high (Fig. 3).

## Hematoxylin–Eosin (HE) staining for iTILs

**Fig. 4:**
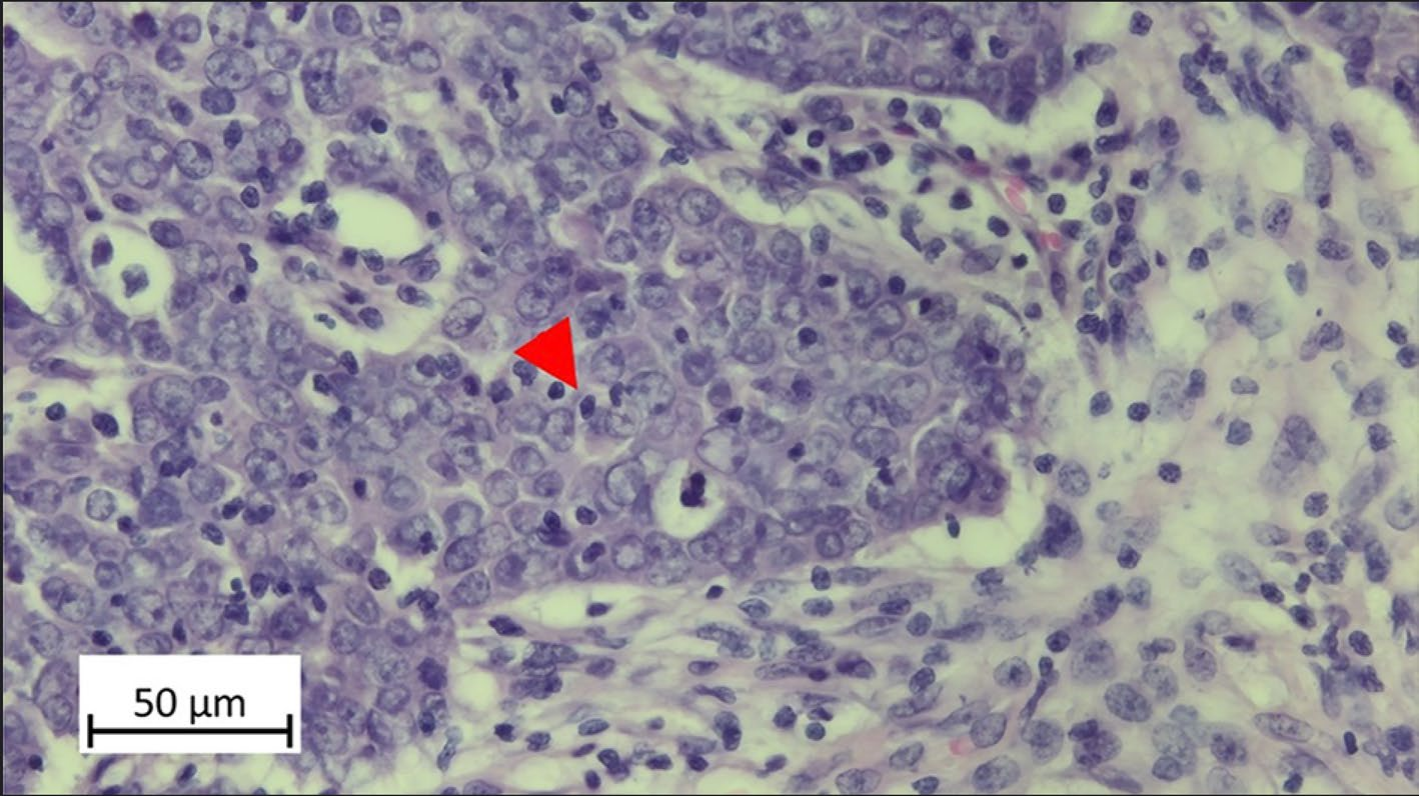
Representative iTILs in OPSCC

Histological sections were stained with hematoxylin–eosin (HE) using the VENTANA BenchMark ULTRA immunostainer (Roche). The slides were classified in a blinded fashion without knowledge of the clinicopathological data by two authors (I.S., M.R.). For the evaluation of intratumoral TILs, the methodology described by De Keukeleire et al. [3] was applied. In the statistical analysis, as in De Keukeleire, an iTILs score of < 5% was categorized as low, and a score of ≥ 5% as high (Fig. 4).

## HPV typing by PCR

For the PCR the DNA was extracted from the paraffin-embedded samples and the QIAamp DNA FFPE Tissue Kit (Qiagen) was used in accordance with the manufacturer’s instructions. DNA integrity was assessed through β-globin gene PCR [27]. HPV DNA was detected using the Anyplex™ II HPV28 Detection Assay (Seegene) and BSGP-5+/6 + PCR [19], and the latter PCR product was typed using a bead-based protocol on a Luminex Lx200 as published [18, 20]. For normalization of mRNA levels the detection of the housekeeping gene β-Actin was used.

A tumor was considered HPV positive if HPV DNA was detected and the immunohistochemical staining for p16 was positive.

### Statistical analysis

Statistical analyses were performed using the SPSS Base System (version 29; SPSS Inc., Chicago, IL, USA). The correlation between PD-L1, VCP/p97, iTILs and HPV status and clinical data was analyzed by chi-square test or Fisher’s exact test. Overall survival time and relapse-free survival time related to PD-L1, VCP/p97 and iTILs- with and without stratification by HPV status- were determined using the Kaplan-Meier method. Univariate analyses were conducted using the log-rank test.

## Results

### VCP/p97 expression in OPSCC

Low VCP/p97 expression (staining intensity level 0–1) was detected in 32.1% of cases (*n* = 18), while high expression (level 2–3) was observed in 67.9% (*n* = 38) (Table 2). Most OPSCC specimens showed a homogenous staining pattern across the entire tumor section.

**Table 2:** Immunohistochemical results of the OPSCC patients

VCP expression	*N*	%	*N* = 56
High expression (2–3)	38	67.9	
Low expression (0–1)	18	32.1	
PDL-1 TPS			*N* = 56
High (≥ 20)	26	46.4	
Low (1–19)	2	3.6	
Negativ (0)	28	50.0	
PDL-1 CPS			*N* = 56
High (≥ 20)	23	41.1	
Low (1–19)	10	18.8	
Negativ (0)	23	41.1	
iTILs			*N* = 56
High (≥ 5%)	20	35.7	
Low (< 5%)	36	64.3	

## PD-L1 expression in OPSCC

Evaluation of PD-L1 combined positive score (CPS) revealed a CPS of 0 in 41.1% of cases (*n* = 23), a CPS of 1–19 in 18.8% (*n* = 10), and a CPS ≥ 20 in 41.1% (*n* = 23). The PD-L1 TPS value of 0 was found in 50.0% (*n* = 28). A TPS value of 1–19 was observed in 3.6% (*n* = 2), and a TPS value ≥ 20 in 46.4% (*n* = 26) (Table 2).

### iTILs in OPSCC

A low iTILs score was observed in 64.3% of patients (*n* = 36), while a high iTILs score was found in 35.7% (*n* = 20).

### HPV status in OPSCC

In 54 OPSCC samples DNA integrity was sufficient. HPV was detected in 48.1% of cases (*n* = 26), while 51.9% (*n* = 28) were HPV-negative (Table 1). All HPV-positive tumors, confirmed by PCR, also showed positive p16 immunostaining. Conversely, all HPV-negative tumors were negative in both PCR and p16 staining. All samples showed HPV 16.

### Correlation of PD-L1 expression and VCP/p97 expression

A significant association was observed between low PD-L1 TPS and high VCP/p97 expression (*p* = 0.025). No significant correlation could be shown between PD-L1 CPS and VCP/p97 (*p* = 0.140).

### Correlation of iTILs and VCP/p97 expression

A significant association was observed between low iTIL density and high VCP/p97 expression (*p* = 0.015).

### Correlation of PD-L1 with HPV-status and clinical data

There was no significant association between PD-L1 TPS and PD-L1 CPS and T-, N-, M-status, Grading and AJCC classification. Furthermore, there was no correlation between PD-L1 TPS and PD-L1 CPS and HPV status. Subgroup analyses stratified by HPV status similarly revealed no significant associations.

### Correlation of VCP/p97 with HPV-status and clinical data

VCP/p97 expression showed no significant correlation with T-, N-, M-status, tumor grading, AJCC classification or HPV status. This finding remained consistent in subgroup analyses according to HPV status.

### Correlation of iTILs with HPV-status and clinical data

High iTILs showed a significant association with positive HPV status (*p* = 0.045). No correlation was found with T-, N-, or M-stage, tumor grading, or AJCC classification.

### Survival analysis

The 5-year overall survival rate in the study cohort was 53.4%, and the 5-year recurrence-free survival rate was 60.0%.

No significant association was found between PD-L1 TPS, PD-L1 CPS, iTILs or VCP/p97 expression and 5 year-overall survival (PD-L1 TPS: 40.0% (low) vs. 46.2% (high), *p* = 0.648; PD-L1 CPS: 36.1% (low) vs. 52.2% (high), *p* = 0.769; iTILs: 55.6% (low) vs. 50.0% (high), *p* = 0.838); VCP/p97: 44.4% (level 0–1) vs. 44.1% (level 2–3), *p* = 0.714). Furthermore, there was no effect on recurrence-free survival during the observation period (PD-L1 TPS: 43.7% (low) vs. 62.0% (high), *p* = 0.575; PD-L1 CPS: 50.9% (low) vs. 48.9% (high), *p* = 0.456; iTILs: 64.2% (low) vs. 52.6% (high), *p* = 0.659); VCP/p97: 64.2% (level 0–1) vs. 48.5% (level 2–3), *p* = 0.533). Stratified analyses of HPV-positive and HPV-negative tumors also revealed no significant differences in either overall or recurrence-free survival.

## Discussion

The aim of this study was to analyze VCP/p97 expression in OPSCC and its relationship with PD-L1 and iTILs. Furthermore, the expression and possible prognostic significance of VCP/p97 in OPSCC was examined, as only one study to date has addressed this issue [12].

In our cohort, high VCP/p97 expression was detected in 67.9% of OPSCC cases. In the study by Meyer et al. 52.8% of the OPSCC had a high VCP/p97 expression [12]. Similarly, 67.6% of gingival carcinomas showed equal or higher staining intensity in tumor cells compared to endothelial cells [32], and a high expression level of 66% was reported in a cohort of 50 laryngeal carcinomas [21]. Overall, these findings suggest that VCP/p97 is frequently overexpressed in HNSCC, supporting its potential as a therapeutic target.

Several studies have investigated VCP/p97 expression as a suitable marker for predicting prognosis [23, 29–33]. In prostate cancer, Tsujimoto et al. [23] demonstrated a significant correlation between high VCP/p97 expression and high tumor volume, advanced tumor stage as well as a significantly reduced disease-free survival and overall survival with high VCP/p97 expression. Yamamoto et al. found a significantly worse disease-free and overall survival in patients with non-small cell lung cancer with high VCP/p97 expression [30] and in esophageal cancer an increased rate of lymph node metastases, a greater depth of tumor invasion and a worse disease-free and overall survival with increased VCP/p97 expression [31]. In thyroid carcinomas, high VCP/p97 expression correlated significantly with lymph node metastases and lower disease-free and overall survival [33]. A significantly higher rate of lymph node metastases, an increased depth of tumor invasion and a significantly poor overall and recurrence-free survival was demonstrated in gastric carcinomas [29]. In gingival carcinomas, patients with high VCP/p97 expression showed a significantly reduced 5-year survival. Meyer et al. [12] demonstrated that HPV-negative OPSCC tumor patients with low VCP/p97 have significantly lower 5-year disease-free survival. In our cohort, patients with low VCP/p97 expression also showed better disease-free survival, although the difference was not statistically significant. Sub-analysis stratified by HPV status revealed also no significant prognostic differences. Furthermore, there was no significant correlations between VCP/p97 expression and T-, N-, or M-status, Grading, HPV status, or AJCC stage. Taken together, these findings suggest that VCP/p97 may not serve as a reliable prognostic biomarker in OPSCC.

However, as mentioned above, VCP/p97 could be a therapeutic target in OPSCC. VCP/p97 inhibitors such as CB-5083, CB-5559 and NMS-873 are known. A phase I clinical trial evaluating CB-5339 in acute myeloid leukemia and myelodysplastic syndrome (https://clinicaltrials.gov, study number NCT04402541) has already been conducted. In esophageal squamous cell carcinoma cell lines, treatment with NMS-873 led to dose-dependent reductions in tumor cell proliferation. Moreover, combining radiotherapy with NMS-873 significantly increased tumor cell death compared to radiotherapy alone [9]. Similar effects were observed by Kilgas et al. [5], who investigated the effect of the VCP/p97 inhibitor in bladder cancer using the RT112 mouse model. They showed a significant reduction in tumor growth compared to radiotherapy alone or therapy with radiotherapy and mirin. In breast cancer models (in vitro, mouse and human organoids) the combination of VCP/p97 inhibitors and PARP inhibitors was shown to be more effective than PARP therapy alone [6]. As mentioned above, Wang et al. [28] demonstrated in a colorectal cancer mouse model that combined treatment with a VCP/p97 inhibitor (CB-5083) and anti-PD-1 significantly inhibited tumor growth compared to monotherapy with anti-PD-1 or control groups. The study further revealed upregulation of PD-L1 expression by inhibition of VCP/p97 via the JAK1/STAT3 signaling pathway. Lo et al. [8] demonstrated that the nanoparticle-mediated co-administration of the VCP/p97 inhibitor CB-5083 and the PD-L1-suppressive microRNA miR-142 significantly reduced tumor growth in the Panc-02 pancreatic cancer mouse model compared to treatment with miR-142 alone. However, the specific effect of VCP/p97 inhibition on PD-L1 expression in tumor cells was not directly assessed.

In our study, immunohistochemical analysis showed a significant correlation between high VCP/p97 expression and low PD-L1 TPS. The PD-L1 TPS results from the PD-L1-positive tumor cells divided by the total number of living tumor cells [17]. This observation could suggest a potential regulatory role of VCP/p97 in PD-L1 expression in OPSCC. A significant correlation between VCP/p97 and PD-L1 CPS was not found. By definition positive mononuclear immune cells are also included in the PD-L1 CPS score. The discordance between TPS and CPS correlations may reflect a tumor cell–specific association of VCP/p97 with PD-L1 expression, while immune cell–mediated PD-L1 regulation could involve alternative mechanisms. Notably, high VCP/p97 expression was simultaneously associated with a significantly lower intratumoral iTIL density. This may explain the discrepancy between TPS and CPS correlations and suggest that VCP/p97 is primarily linked to tumor cell–associated PD-L1 expression, while immune cell–mediated regulation appears to be less relevant in the context of low TIL infiltration. These findings support the hypothesis that VCP/p97 not only influences tumor cell–associated PD-L1 expression but may also contribute to an immune-poor tumor microenvironment. Taken together, these findings may point toward a direct role of VCP/p97 in tumor cell–intrinsic immunomodulation, potentially contributing to an immune-poor microenvironment in OPSCC. However, this requires additional investigation.

In addition, further studies on larger collectives and the investigation of the possible regulation of PD-L1 expression by VCP/p97 in OPSCC tumor cell culture are needed. Exploring combination therapies involving PD-1/PD-L1 checkpoint inhibitors and VCP/p97 inhibitors in OPSCC may offer novel therapeutic avenues. Based on the current findings, such combinations could possibly improve efficacy of checkpoint inhibitor therapy. In this regard, further studies are planned.

However, this retrospective study has limitations. Notably, none of the included patients had received prior systemic therapy for HNSCC. Currently, PD-1/ PD-L1 inhibitor therapy is only approved for palliative treatment in HNSCC. In other tumor entities, such as non-small cell lung cancer, these agents are already approved for neoadjuvant use. In HNSCC, the Keynote 689 study (NCT03765918) has recently been completed. This trial investigated the addition of pembrolizumab in neoadjuvant and adjuvant setting to standard-of-care treatment in patients with locally advanced HNSCC intended for curative therapy. The study demonstrated a significant improvement in event-free survival in patients who received pembrolizumab in addition to standard treatment [25]. Based on these findings, it is reasonable to hypothesize that the integration of pembrolizumab into curative treatment regimens become part of future clinical practice.

## Conclusion

In summary, VCP/p97 is frequently overexpressed in OPSCC. The significant correlation between high VCP/p97 expression and low PD-L1 TPS and low iTILs could possibly indicate that VCP inhibitor therapy may improve efficacy of PD-1 checkpoint inhibitor therapy in OPSCC. However, further studies are required.

## Data Availability

Data is provided within the manuscript. The supplementary data supporting the results of this study are available on request from the corresponding author, I.S.
